# Molecular Docking Analysis of Hydroxyclavicol and Eugenol From Betel Leaves Against Outer Membrane Protein (OmpH) of Dialister pneumosintes

**DOI:** 10.7759/cureus.53809

**Published:** 2024-02-07

**Authors:** Ilamaran Varshan, Sathish Sankar

**Affiliations:** 1 Department of Microbiology, Saveetha Dental College and Hospitals, Saveetha Institute of Medical and Technical Sciences, Saveetha University, Chennai, IND

**Keywords:** molecular docking, dialister pneumosintes, universal health, eugenol, hydroxychavicol

## Abstract

Introduction

*Dialister pneumosintes* is an obligate anaerobic non-spore-forming Gram-negative bacilli. As a part of polymicrobial film, the activated virulence factor causes oral diseases like gingivitis and periodontitis. Decreased susceptibility of clinical strains of *D. pneumosintes* to different antibiotics including piperacillin and metronidazole raises concerns. There has been significant interest in the utility of plant phytocompounds as potent antibacterial agents.

Aim

The study aimed to look at the potential of two phytocompounds, eugenol and hydroxychavicol, for their ability to inhibit outer membrane protein (OmpH) of *D. pneumosintes* using computational tools.

Results

The study showed effective inhibition of the OmpH of *D. pneumosintes* by both eugenol and hydroxychavicol. The high probability to be active (Pa) value indicated the probability of true positive for the tested compounds for their predicted biological activity. There was strong reciprocity between the drug-likeliness and its binding affinity for the target protein, indicating an inhibitory nature.

Conclusion

The tested phytocompounds hydroxychavicol and eugenol showed potential inhibition of the OmpH protein of *D. pneumosintes* indicating its potential use as inhibitory compounds of the pathogen and future directions for the treatment of periodontitis and gingivitis.

## Introduction

*Dialister pneumosintes* is an obligate anaerobic Gram-negative bacillus. They are non-motile, non-spore-forming and do not ferment any carbohydrates. These non-lactose-fermenting *D. pneumosintes* are a natural part of the bacterial flora found in the pharynx, colon, oral cavity, and vagina [1,2]. First identified as *Bacterium pneumosintes, *they have been reported in endodontic infections. It has recently been shown that this microbe can cause infection through a hematogenous route in different parts of the body resulting in maxillofacial and periodontal tissues or soft tissue abscesses infections. Biofilm formation of this bacteria stimulates the virulence factor and aids in the development of dental diseases including periodontitis and gingivitis [1,3]. Being one of the neglected diseases, especially in developing countries and lack of appropriate diagnostic methods and treatment modalities, the risk of infections in immunocompromised individuals is of major concern. Apart from the periodontal infections, involvement of the head, neck and respiratory tract warrants the need for appropriate therapeutic options.

In recent years, there has been an increasing interest in the use of phytocompounds for treating dental diseases. Dental pathogens, such as *D. pneumosintes*, *Porphyromonas gingivalis*, *Treponema denticola*, *Tannerella forsythus*, and* Aggregatibacter actinomycetemcomitans* are either difficult to grow in media or difficult to identify using biochemical methods. Hydroxychavicol and eugenol are polypropanoid compounds present in plants that have been shown to have potential uses against dental diseases. Eugenol is also found in clove, *Eugenia aromatica*, and exhibits anti-inflammatory, antibacterial, antioxidant, analgesic, and local analgesic properties [4]. 

Molecular docking has grown to be a significant step in the drug discovery procedure. Since its inception in the 1980s, docking has gained popularity in both academic and industrial contexts due to improvements in methods developed since then, including the development of more accessible small molecule and protein structures and advances in computer hardware power. The ways in which docking is employed to support the various drug discovery tasks have evolved over time. Docking was first created and used as a stand-alone technique, but it is now primarily used in integrated workflows in conjunction with other computational techniques.

In the present study, the authors aimed to identify the inhibitory effect of hydroxychavicol and eugenol against the outer membrane protein of *D. pneumosintes *through a bioinformatic approach.

## Materials and methods

Protein modeling

The three-dimensional (3D) protein structure of outer membrane protein H (OmpH) of *D. pneumosintes* was not available in the protein databank. Hence, the protein was modeled using SWISS-MODEL online server program (https://swissmodel.expasy.org/) [5]. The SWISS-MODEL template has been used as templates using Basic Local Alignment Search Tool (BLAST) and using HHblits, a search tool that utilizes iterative pairwise comparison of profile hidden Markov models. ProMod3 program was used to build models based on target-template alignment. The model copies the coordinates from the template that are conserved between the target and the template. Phytochemicals identified from betel, namely hydroxychavicol and eugenol, were selected for the study. 

Absorption, distribution, metabolism, elimination, and toxicity (ADMET)

Interaction of the drug including pharmacological effects that include their effects on cells, organs and systems, their enzymatic activity, function and specific biochemical action of the drug, interplay with metabolizing enzymes and drug transporters during absorption and assimilation, pharmacogenomics that includes the interaction with gene expression and subsequent protein synthesis, possible adverse effects and side effects were predicted using Passonline web server (http://www.way2drug.com/passonline). Simplified Molecular Input Line Entry System (SMILES) of each drug was fed as the input for each compound. Ten interactions with the highest Pa value were selected for analysis. 

Pa, which denotes the probability to be active, is an estimate of the probability of the compound belonging to the sub-class of active compounds resembling the established set of actives in the server training set. Similarly, Pi is the probability of being inactive that is estimated by the server. The physiochemical properties including topological polar surface area (TPSA) score, GI absorption, cytochrome inhibition, lipophilic nature, water solubility, pharmacokinetics and drug-likeliness were assessed using the SwissADME online server program. SMILES of each compound was used as the input. Optimal docking areas were calculated using molsoft online server (http://www.molsoft.com). The output was viewed using ICM-browser.

Molecular docking 

The 3D structures of all the ligands were obtained from PubChem database (https://pubchem.ncbi.nlm.nih.gov/) and verified with DrugBank (https://go.drugbank.com/k). The file formats of the chosen ligands were then converted into protein data bank (PDB) format utilizing OpenBabel program and set for docking using AutoDock. The receptor and the ligand were fed as ".pdb" file and the resulting interactions with the docking score were recorded. The protein structure of OmpH was prepared using the AutoDock program 1.5.6. By varying the values of the grid parameters x, y, and z coordinates, the binding site for the protein-ligand interaction of the target Spike protein from many versions was identified using grid box creation. Based on the docked complex, the binding score, hydrogen bonding, and hydrophobic interaction between the target protein and docked ligands were evaluated. The ligands deemed most efficient against the target protein were those with the lowest E score. The hydrogen bond interaction between phytocompounds (ligands) and the target protein in a docked complex, as well as the length of the bond and the interactions between amino acid residues, were examined using the PyMol visualization tool.

## Results

ADMET properties

The biological activity of the hydroxychavicol and eugenol was tested using PassServer online program. The analysis revealed two important parameters: Pa, which is the probability to be revealed, and Pi, which is the probability of not being revealed. The Pa and Pi values varied from 0 to 1 and were independent of each other. These two factors were taken into account to identify the biological activity spectrum associated with the compound with desirable and non-desirable values. The high Pa value indicated the probability of true positive for the tested compounds for their predicted biological activity. In our study, among the list of activity spectra predicted, the most common 10 biological activities for Pa and Pi of hydroxychavicol (Table 1) and eugenol were selected (Table 2).

**Table 1:** Biological activity of hydroxychavicol Pa: Probability to be active; Pi: Probability to be inactive

Pa	Pi	Activity
0.930	0.001	Carminative
0.925	0.001	Steroid N-acetylglucosaminyltransferase inhibitor
0.928	0.004	Aspulvinone dimethylallyltransferase inhibitor
0.900	0.005	Chlordecone reductase inhibitor
0.894	0.005	Mucomembranous protector
0.897	0.012	Membrane integrity agonist
0.883	0.006	Antieczematic
0.875	0.006	G-protein-coupled receptor kinase inhibitor
0.875	0.006	Beta-adrenergic receptor kinase inhibitor
0.866	0.004	CYP2E1 substrate

**Table 2:** Biological activity of eugenol Pa: Probability to be active; Pi: Probability to be inactive

Pa	Pi	Activity
0,941	0,001	Carminative
0,937	0,004	Aspulvinone dimethylallyltransferase inhibitor
0,902	0,005	Chlordecone reductase inhibitor
0,881	0,005	Feruloyl esterase inhibitor
0.878	0.003	Antimutagenic
0.873	0.004	Caspase 3 stimulant
0.873	0.004	JAK2 expression inhibitor
0.868	0.008	Antieczematic
0.863	0.004	Linoleate diol synthase inhibitor
0.856	0.004	CYP2E1 substrate

The ADMET properties that included chemical absorption, distribution, metabolism, excretion and toxicity were predicted for the two compounds. This analysis proved the ability of the compounds as appropriate as therapeutic agents. The prediction was used to identify the drug-likeliness properties of the compound. Several factors including the factors determining the pharmacokinetics were included in the program (Table 3). After hydroxychavicol was subjected to an ADME screening, it was discovered that there was a strong reciprocity between the drug-likeliness and its binding affinity for the target protein, indicating an inhibitory nature. This information was further supported by a molecular docking analysis.

**Table 3:** Important ADMET parameters of the compounds TPSA: Topological polar surface area; Log Po/w: Arithmetic mean of estimation of lipophilicity of molecules; GI: Gastrointestinal; ADMET: Absorption, distribution, metabolism, elimination, and toxicity

Parameters	Hydroxyclavicol	Eugenol
TPSA	29.46	40.46Å^2^
Consensus Log P_o/w_	2.25	1.53
Water solubility	Soluble	very soluble
GI absorption	High	High
Inhibitors of CYP1A2, CYP2C19, CYP2C9, CYP2D6, CYP3A4	no	no
Lipinkski (drug likeliness)	yes, 0 violation	yes, 0 violation
Bioavailability score	0.55	0.55
Synthetic accessibility	1.58	1.35

Molecular docking 

Using biopredicta tools, the target protein's protein databank file was downloaded and examined for any structural flaws. With the assistance of neighboring residues, incomplete atoms or residues were altered or transformed. Protein chains that weren't desired were eliminated from the structure by choosing the chain and removing it. With the use of Biopredicta tools and coordinates, the target protein was examined for parameters including intertwined residues, geometric check (local) and Ramachandran plot using PROCHECK server. The bond length was 15%, the bond angle was 25%, and the bond length was 15% for the local geometry check. In order to reduce mistakes in the interactions between the protein and ligand, the protein was ultimately tuned using the calculated forcefield option. 

**Figure 1:**
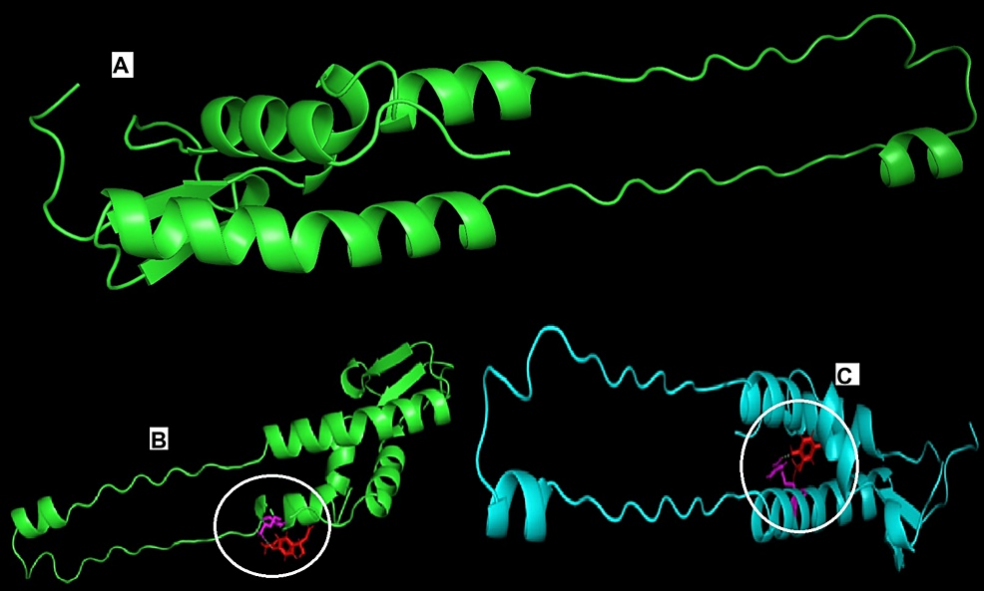
Docked complex - hydroxychavicol and eugenol The three-dimensional structure of OmpH protein of *D. pneumosintes* (A). Docking of hydroxychavicol (B) and eugenol (C) with the protein is indicated in white circles.

Molecular docking was carried out with hydroxychavicol and eugenol on OmpH protein of *D. pneumosintes*. The resulting interactions were viewed, annotated and analyzed using PyMol program (Figure 1). Using the AutoDock program, the molecular docking experiments with the OmpH protein were conducted using molecules that met Lipinski's rule of five. The results were interpreted based on the cumulative docking score, the number of interactions with active site residues, the number of H-bonds generated, and the bond length (Table 4). The visualization program PyMol was used to monitor the interactions. The target protein's docking score was lowest for eugenol. The pharmacological potential effects of the compounds using molecular docking showed the effect of binding with the bacterial outer membrane protein. This indicates the potential effect of the compound to act as a drug candidate for the inhibition of bacteria. The in silico prediction of binding efficacy demonstrated its efficacy for more advanced drug testing.

**Table 4:** Binding interactions of the protein with the ligand LYS: Lysine; ARG: Arginine; E-value: Binding energy

Phytochemicals	PubChem ID	Binding residues	E-value
Hydroxychavicol	71596738_	LYS-68	-157.62
Eugenol	3314	ARG-117	-169.59

Using the OpenBabel computational program, the two-dimensional (2D) structures of the phytocompounds (ligands) were transformed into an ideal 3D form. The chosen receptors were docked with the phytochemical ligands. In the current investigation, the ligands demonstrated distinct types of interactions with certain receptor proteins. During docking analysis, the protein-ligand interactions were evaluated, with a focus on the docked poses that exhibited substantial dock scores. Most of the targets had docking scores that were somewhat higher than co-crystal ligand scores, indicating that the compounds were better able to bond to each other than the co-crystal ligands. The ligands that bind similarly to the reference ligands' orientation were determined using the molecular docking scores. In contrast to the reference ligand, the majority of phytocompounds, or ligands, produced excellent docking postures. The deep docking of selective ligands inside the binding pocket area indicates that their shapes are complementary to those of the reference ligands. An analysis of the ligands' Pi stacking, hydrogen bonding, and hydrophobic interactions with receptor proteins produced some new data (Table 4). 

## Discussion

The present study looked at the potential of two major phytocompounds, namely hydroxychavicol and eugenol, for their inhibitory activity against dental pathogen *D. pneumocintes* using a bioinformatic approach. The pharmacological properties of the two compounds including their biological activity, and ADMET properties were analyzed for their use as a drug for diseases caused by *D. pneumosintes*. It has been suggested that *D. pneumosintes* may be a novel periodontopathic species. This investigation assessed the microbial predominance in subgingival biofilm and saliva from participants with various periodontal diseases.

Only three percent of the saliva samples had *D. pneumosintes* identified, compared to 47.8% of the biofilm samples. In locations with both periodontal health and infection, CP patients showed a noticeably higher mean prevalence of this species. There were noteworthy correlations found between the frequency of *D. pneumosintes* and the depth of the pocket, attachment loss, and bleeding upon probing with statistical significance (with p p-value less than 0.005). Results support the hypothesis that *D. pneumosintes* causes periodontitis [6]. Even though *D. pneumosintes* has been found in deep periodontal pockets, not much is understood about how the bacterium and harmful periodontal disease are related.

Due to the positive effects on several important diseases, phytocompounds and medicinal herbs which were utilized in traditional old medicine are currently being examined more and more in clinical and experimental settings. Phytotherapy, like the pharmaceutical sector, is interested in identifying and characterizing the cellular and molecular therapeutic targets of plant components via the use of nano-based delivery methods. The primary phenolic component of clove essential oil, eugenol, is a highly adaptable phytochemical with a wide variety of medicinal uses, some of which have been well-studied. These include anti-inflammatory, antioxidant, and anticarcinogenic actions. The last 10 years have seen a surge in research on the function of phytochemicals as bioenergetic and metabolism modulators due to our growing awareness of the role of mitochondria as essential organelles in the pathophysiology of noncommunicable illnesses [7].

There have been two documented incidences of *D. pneumosintes*-related brain infections in previously healthy individuals. The bacterium was directly obtained from a brain abscess in the second patient and from the first patient via blood culture. It was believed that the illness in both patients had a dental or nasopharyngeal origin [8]. After receiving antimicrobial therapy and surgical debridement, the patient’s prognosis improved. Following partial sequencing and in vitro amplification of the 16S rRNA gene, two strains were identified as *D. pneumosintes*. Nevertheless, the bacteria could not be identified by conventional biochemical testing. Apart from inducing opportunistic and periodontal infections, *D. pneumosintes*, which is present in mixed flora, has the potential to function as a clinically significant pathogen, particularly in the brain [6,9,10].

Eugenol has three main properties: it inhibits the cyclooxygenase2 enzyme, selectively binds to the capsaicin receptor in the epithelium to provide analgesic effects, and has antibacterial action against both gram-positive and gram-negative bacteria. By blocking N-methyl-d-aspartate (NMDA), which is implicated in pain sensitivity, eugenol also has anti-nociceptive characteristics. In order to lessen discomfort, eugenol is often used topically in conjunction with lignocaine or prilocaine. The deep heart-shaped leaves of *Piper betle*, also known as betel leaves, are a natural source of hydroxychavicol (4-allyl-cat-epil, 1-allyl-3,4-dihydroxybenzene), a significant phenolic component that has historically been utilized as a mouth freshener in eastern Asian native countries. It has been shown to possess the ability to trigger apoptosis in cells via the induction of oxidative stress, depletion of glutathione (GSH), and modulation of the cell cycle [11]. By stimulating the body's innate immunological response, hydroxychavicol also has anticancer effects. and trigger the mitochondrial signaling pathway and P53 activation to cause cell apoptosis. By generating reactive oxygen species (ROS), hydroxychavicol has been shown in some studies to have anti-inflammatory, antioxidant, anti-nitrosation, anti-mutagenic, and anti-carcinogenic properties against carcinogens and mutagens. Betel leaves are rich in eugenol, a naturally occurring phenol [12]. Numerous more therapeutic plants, such as bay leaves, cloves, and cinnamon, also contain the phytochemical. This substance is used topically as an antibacterial and an irritant in dental treatments containing zinc oxide for root canal therapy [13-15]. It has been shown that this substance has analgesic, neuroprotective, antipyretic, antioxidant, and antifungal qualities. Studies have shown that interferes with action potential conduction, albeit the precise mechanism of action is unclear. Many over-the-counter toothache remedies include eugenol, which is not authorized. According to research, eugenol has a significant effect on the biofilms of clinical strains of methicillin-resistant Staphylococcus aureus (MRSA) and methicillin-susceptible Staphylococcus aureus (MSSA). Eugenol killed the bacteria in both MRSA and MSSA biofilms equally well, hindered the production of new biofilms, broke down cell-to-cell connections, and detached the ones that already existed. Eugenol may thus be utilized to treat or completely remove infections brought on by *S. aureus* biofilms [16]. There are no appropriate therapeutic strategies available to treat infections caused by biofilm producers while the antibiotic resistance further hampers the treatment modalities. In this scenario, alternative therapies such as use of effective phytocompounds such as eugenol and hydroxychavicol could help act against biofilms or can aid antibiotic action.

Leaves of *P. betle* are extensively grown in Malaysia, India, Indonesia, and Thailand. Because of their high phenolic content, they have been used for centuries in traditional medicine owing to their therapeutic qualities, which include antibacterial, antifungal, antiproliferative, antioxidant, and anti-inflammatory effects. Hydroxychavicol is one of the main ingredients of *P. betle* leaves, they have been shown to have an antiproliferative effect at micromolar levels on a variety of cancer cell lines from diverse sources while sparing healthy cells. In vitro, studies have identified a multitude of probable pathways behind hydroxychavicol's chemopreventive actions against cancer cell lines. These included investigations on cancer cell lines from the prostate, glioma, breast, and colorectal malignancies, as well as studies on Ehrlich ascites carcinoma cells in Swiss albino mice and a chronic myeloid leukemia (CML) mouse model [3,17]. 

The allylbenzene hydroxychavicol has drawn a lot of interest because of its anticancer qualities. Using cytotoxicity and in silico analyses, hydroxychavicol from *P. betle* L. was measured, and purified, and its anticancer potential was projected. The physicochemical property recommendations of Lipinski's Rule of Five were fulfilled by the ADMET properties of eugenol and hydroxychavicol utilizing SwissADME, guaranteeing its drug-likeness behavior. Using 16 different cancer targets, hydroxychavicol's interaction with them was validated by molecular docking experiments [18-20]. The anticancer competence of hydroxychavicol was also shown in the in vitro MTT test using bone cancer cell lines (MG63), highlighting the need to develop the molecule as a medication to treat different kinds of malignancies.

The study however had limitations including lack of experimental evidence in experimental animals. The pathogen is difficult to cultivate, hence an infection model could be developed for further investigations and to prove the use of these phytocompounds in therapy.

## Conclusions

*D. pneumosintes*, as a part of normal flora, causes periodontal infections and is of rising concern. Due to its neglected status, particularly in underdeveloped nations where treatment options and diagnostics are inadequate, immunocompromised persons are at a significant risk of infection. In addition to periodontal diseases, the head, neck, and respiratory tract involvement necessitates the use of suitable treatment alternatives. The study showed the potential use of hydroxychavicol and eugenol in treating infections caused by *D. pneumosintes*. Molecular docking experiment was employed to investigate the ability of the drug to bind to the outer membrane protein of the pathogen. The molecular docking technique may be used at different stages of the drug discovery and design process for cancer and infectious disorders. This technique may be used to match a lead molecule to a protein target, or conversely, it can be used to match a protein target to a query ligand.
